# Implementation of the GLIM (Global Leadership Initiative on Malnutrition) Criteria in Gastrointestinal Oncology: A Narrative Review of Systemic Impact and the Role of Dietitians

**DOI:** 10.7759/cureus.86826

**Published:** 2025-06-26

**Authors:** Keita Ishido, Mio Nakashima, Saseem Poudel, Zen Naito, Akitaka Motoyoshi, Kaito Sano, Satoshi Hirano

**Affiliations:** 1 Surgery, Asahikawa City Hospital, Asahikawa, JPN; 2 Gastroenterological Surgery II, Hokkaido University Faculty of Medicine, Sapporo, JPN; 3 Nutrition, Japanese Red Cross Asahikawa Hospital, Asahikawa, JPN

**Keywords:** clinical dietitian, gastrointestinal cancer, glim criteria, multidisciplinary care, nutritional assessment

## Abstract

Malnutrition is a common yet frequently underrecognized condition among patients with gastrointestinal cancers, significantly impacting treatment tolerance, postoperative recovery, and long-term outcomes. This narrative review explores the clinical implementation of the Global Leadership Initiative on Malnutrition (GLIM) criteria in gastrointestinal oncology, with a particular focus on its impact on multidisciplinary workflows and the evolving role of clinical dietitians. A structured literature search was conducted using PubMed to identify English-language publications from 2018 to 2025 that included the terms “GLIM,” “malnutrition,” “gastrointestinal cancer,” “nutritional assessment,” and “oncology.” Fifty-three articles were selected for inclusion based on their relevance to GLIM criteria implementation and interdisciplinary care models in oncologic settings. In clinical practice, the GLIM framework has elevated nutrition care from an ancillary task to a core component of oncology management. The adoption of GLIM criteria encourages interprofessional collaboration, involving surgeons, oncologists, nurses, and dietitians. It also necessitates the use of tools such as bioelectrical impedance analysis and computed tomography to assess muscle mass, as well as laboratory markers of inflammation, further reinforcing the technical competencies required of dietitians. However, barriers remain. Implementation is hindered by limited training, workflow inconsistencies, time constraints, and disparities in access to diagnostic tools, particularly in resource-limited settings. Additionally, institutional variability and lack of integration into reimbursement systems pose systemic challenges. To support sustainable adoption, many institutions have established structured referral pathways, interdisciplinary training initiatives, and electronic health record algorithms for GLIM-based assessment. These efforts are crucial for overcoming barriers and standardizing care. Furthermore, GLIM-based protocols have improved patient engagement and empowered patients to participate more actively in their nutritional care, especially through the use of standardized tools like the Patient-Generated Subjective Global Assessment. Looking forward, further research is needed to validate simplified diagnostic methods, improve equity in access, and assess long-term impacts on survival and quality of life. Policy reforms, including the recognition of GLIM-based malnutrition in coding systems, could enhance institutional incentives for implementation. Ultimately, the GLIM criteria provide not only a diagnostic tool but also a foundational framework for advancing equitable, interdisciplinary, and patient-centered nutrition care in oncology.

## Introduction and background

Malnutrition is a prevalent and consequential condition among patients with gastrointestinal cancers, significantly impacting postoperative recovery, tolerance to chemotherapy, length of hospital stay, and overall survival [1-4]. The prevalence of malnutrition in this population has been reported to exceed 40%, underscoring the need for systematic identification and intervention [2,5].

Gastrointestinal cancers, particularly gastric and colorectal malignancies, often lead to malnutrition due to tumor burden, gastrointestinal symptoms, or treatment-related effects. These cancers are among the most common globally and frequently impair nutrient intake and absorption, making nutritional screening and intervention essential. Despite its clinical importance, malnutrition often remains underdiagnosed and inconsistently managed due to the lack of standardized diagnostic criteria across institutions and disciplines [6].

To address this gap, the Global Leadership Initiative on Malnutrition (GLIM) was established in 2016 by a coalition of major clinical nutrition societies. In 2018, GLIM proposed a consensus-based diagnostic framework that combines two core components: phenotypic criteria (such as weight loss, low body mass index [BMI], and reduced muscle mass) and etiologic criteria (including reduced food intake or assimilation and the presence of inflammation) [7,8]. This dual-component approach aims to harmonize malnutrition diagnosis across clinical settings and patient populations. Unlike tools such as the Subjective Global Assessment (SGA), Mini Nutritional Assessment (MNA), or Malnutrition Screening Tool (MST), which primarily serve as screening instruments, the GLIM criteria offer a two-step diagnostic framework combining clinical symptoms with objective measurements of body composition and inflammation [9-12].

Since its publication, the GLIM criteria have gained international traction, with increasing numbers of validation studies and implementation efforts across medical specialties, particularly in oncology. Compared to traditional tools such as the SGA or MNA, the GLIM criteria provide a more objective and universally applicable framework for diagnosis [9-12]. Early evidence also suggests that the GLIM criteria-defined malnutrition is strongly associated with adverse clinical outcomes, including increased postoperative complications, prolonged hospitalization, and reduced survival in patients with gastrointestinal cancers [13-16].

Nevertheless, the transition from diagnostic consensus to routine clinical adoption remains incomplete. Barriers such as limited provider training, lack of standardized implementation pathways, and resource disparities across institutions have hindered widespread uptake [17,18]. Furthermore, the integration of the GLIM criteria into multidisciplinary workflows, electronic health records (EHRs), and patient-centered care models remains variable and underexplored.

This narrative review aims to synthesize current evidence on the implementation of the GLIM criteria in gastrointestinal oncology. Specifically, we examine its clinical integration, impact on dietitian practice, effects on patient outcomes, and ongoing challenges.

## Review

Methods

This review focuses on gastric and colorectal cancers, as they represent the two most common gastrointestinal malignancies and have the most extensively documented use of the GLIM criteria in clinical nutrition research. We performed a structured literature search using PubMed for English-language articles published between 2018 and 2025. The search was centered on the terms “GLIM,” “malnutrition,” “gastrointestinal cancer,” “nutritional assessment,” and “oncology.” After screening titles and abstracts, 54 publications were included in this review based on relevance to oncology-focused GLIM criteria implementation and interdisciplinary care. 

The PubMed search included the following terms: “GLIM,” “malnutrition,” “gastrointestinal cancer,” “nutritional assessment,” and “oncology.” Filters were applied for English language and human studies, with a publication date range of 2018 to 2025. Studies were included if they examined the implementation, validation, or clinical integration of the GLIM criteria in patients with gastric or colorectal cancer. We excluded articles that did not focus on gastrointestinal oncology or that only referenced the GLIM conceptually without applied implementation. No formal risk-of-bias assessment was performed, consistent with the methodology of narrative reviews.

Implementation of GLIM criteria in clinical practice

The integration of the GLIM criteria into clinical oncology settings has reshaped how nutrition is addressed across multidisciplinary teams. Traditionally, nutritional screening was handled primarily by dietitians using tools like the MST or NRS-2002. In contrast, the GLIM criteria require a broader interprofessional effort, where physicians, nurses, dietitians, and allied health professionals collaborate not only to identify malnutrition risk but also to confirm diagnosis using a two-step process involving phenotypic and etiologic components [19-21]. This shift has required oncology teams to restructure workflows to accommodate diagnostic steps such as body composition assessment and evaluation of inflammatory markers, tasks that extend beyond conventional screening.

One of the most significant institutional changes has been the formalization of nutrition screening and referral systems. Many cancer centers have embedded nutritional risk screening into preoperative checklists or chemotherapy planning protocols. When a patient is identified as at risk by preliminary tools, GLIM-based assessment is triggered automatically, resulting in timely referral to clinical dietitians [20-22]. This systematization ensures that nutritional deficits are not overlooked and that appropriate interventions can be initiated early in the care trajectory, reducing treatment delays and mitigating risk.

In tandem with process changes, technological advancements have facilitated GLIM implementation. Hospitals equipped with electronic medical records (EMRs) have begun integrating algorithm-based prompts that signal clinicians when GLIM criteria should be applied [23]. These EMR-integrated workflows not only support documentation but also enable real-time alerts, allowing interdisciplinary teams to coordinate interventions quickly and effectively. Some institutions have reported improved patient throughput and reduced length of stay after implementing these digital workflows [24].

Beyond structural and technological shifts, a notable cultural change is also evident. Malnutrition is no longer viewed as a peripheral issue, but as a condition that substantially affects treatment efficacy, complication rates, and survival outcomes. This recognition has elevated the status of nutrition care and positioned clinical dietitians as essential contributors to treatment planning alongside surgeons, oncologists, and pharmacists [25]. Institutions that have embraced GLIM criteria have expanded their Nutrition Support Teams, conducting regular multidisciplinary case conferences and integrating nutritional care into Enhanced Recovery After Surgery (ERAS) protocols, particularly in gastrointestinal cancer cases [19,26].

However, implementation is not without challenges. Hospitals with successful GLIM adoption often credit clinical champions - healthcare professionals who advocate for nutrition screening and lead the development of tailored implementation protocols. These individuals also play a pivotal role in providing hands-on training, fostering interprofessional buy-in, and overseeing ongoing quality improvement initiatives [27,28]. Without this leadership and infrastructure support, GLIM criteria can remain underutilized despite their clinical value.

Impact on dietitians’ role and workflow

The implementation of the GLIM criteria has profoundly transformed the role of dietitians in gastrointestinal oncology, extending their responsibilities beyond traditional nutritional counseling into active diagnostic and decision-making roles. Historically, dietitians have focused on individualized meal planning, dietary education, and supplementation guidance. However, the two-step structure of the GLIM criteria, which requires phenotypic confirmation (such as weight loss or reduced muscle mass) and identification of etiologic contributors (such as decreased intake or inflammation), has positioned dietitians at the core of the malnutrition diagnostic process [5,29,30].

Dietitians are now directly involved in interpreting clinical and laboratory data related to body composition, dietary intake, and inflammation. For instance, assessing muscle mass, a key phenotypic criterion, often necessitates the use of modalities like bioelectrical impedance analysis (BIA), computed tomography (CT), or dual-energy X-ray absorptiometry (DXA), which require specialized knowledge to interpret accurately [31]. In many settings, dietitians collaborate with radiologists or oncologists to evaluate these metrics, contributing to the formulation of the final malnutrition diagnosis. Similarly, understanding the nuances of inflammation markers, particularly in patients undergoing chemotherapy or those with chronic disease, demands advanced clinical reasoning and familiarity with evolving diagnostic thresholds.

The GLIM criteria have also prompted significant institutional changes that restructure dietitians' integration into patient care. Hospitals implementing GLIM often establish structured referral pathways, ensuring that once malnutrition risk is flagged by preliminary screening, patients are promptly evaluated using GLIM-based diagnostic processes led or co-managed by dietitians [20,21]. This shift allows for timely nutritional interventions, which have been shown to improve clinical outcomes such as reduced postoperative complications, improved chemotherapy tolerance, and lower readmission rates [26].

Moreover, updated clinical guidelines now endorse the inclusion of registered dietitians in treatment planning and documentation, recommending that they lead the development and monitoring of comprehensive nutritional care plans following GLIM-based diagnosis [32]. These plans typically include individualized energy and protein prescriptions, micronutrient support, monitoring schedules, and patient education on disease-specific nutrition management. As a result, dietitians are increasingly regarded not only as care providers but as diagnosticians with clinical authority within multidisciplinary teams [33].

To meet these demands, dietitians must acquire advanced competencies not previously considered core to their training. These include interpretation of systemic inflammation, familiarity with imaging and anthropometric data, and the ability to synthesize EHR data for real-time clinical decisions [29,34]. However, the increased complexity of this role presents logistical challenges in busy oncology centers, where dietitians face high patient volumes and limited time per consultation [35].

Recognizing this need, professional societies and hospital systems have launched initiatives to support dietitians’ upskilling. Continuing education programs, certification modules, and practice guidelines focused on GLIM implementation have been introduced to standardize clinical competencies across institutions [6,36]. In some cases, interdisciplinary workshops have facilitated team-based learning, improving communication between dietitians, physicians, and nurses, and helping embed nutrition into routine oncologic care.

Ultimately, the GLIM criteria have redefined the scope and visibility of dietitians in cancer care. Their expanded diagnostic and clinical roles underscore the importance of positioning dietitians as integral players in achieving evidence-based, personalized, and timely nutritional interventions for patients with gastrointestinal malignancies.

Patient-centered outcomes and support systems

The adoption of the GLIM criteria in gastrointestinal oncology has had a measurable impact not only on clinical workflows but also on patient-centered outcomes, underscoring the role of standardized nutritional assessment in improving the quality and continuity of care. By enabling early and structured identification of malnutrition, the GLIM framework has facilitated timely nutritional interventions that contribute to improved functional status, reduced complications, and enhanced patient engagement across the cancer care continuum [37-39].

In clinical practice, the use of GLIM-based protocols has been associated with tangible improvements in treatment tolerance and recovery. For instance, patients identified early as malnourished, particularly those with gastric or colorectal cancer, benefit from tailored dietary interventions that preserve lean body mass and improve oral intake during chemotherapy or surgical recovery [37]. Multiple studies have reported associations between GLIM-guided care and favorable clinical outcomes, including reduced length of hospital stay, lower rates of postoperative infections, and improved tolerance to chemotherapy agents [38,40,41]. In colorectal cancer, GLIM-based nutritional care has been linked to improved functional independence and decreased hospital readmissions during the post-discharge period [42].

The implementation of GLIM criteria also enhances patient agency and engagement. By incorporating patient-centered tools, such as the Patient-Generated Subjective Global Assessment (PG-SGA) and structured dietary logs, patients are invited to participate actively in their nutritional assessment. This fosters greater awareness of the impact of nutrition on treatment success and empowers patients to adhere to dietary recommendations [40]. This empowerment is particularly important in survivorship settings, where long-term self-management of nutrition is critical to maintaining functional health and preventing recurrence-related malnutrition [41].

To support this shift toward structured and continuous nutritional care, many institutions have introduced supplementary systems tailored to the GLIM criteria. For example, routine outpatient screening programs have been implemented to detect nutritional risk prior to surgery or chemotherapy initiation, allowing for preemptive intervention [37,43]. Education materials aligned with GLIM diagnostic components, such as guidance on protein intake or anti-inflammatory nutrients, have been standardized and integrated into patient education sessions [6,34]. These materials serve to reinforce clinical messaging and provide patients with consistent, evidence-based nutritional information that they can apply outside the clinical setting.

Furthermore, community-anchored follow-up programs have emerged to bridge inpatient and outpatient nutrition care. These include telehealth consultations, home-visit dietetic services, and collaborations with primary care providers to ensure continuity of GLIM-based monitoring after discharge [39,44]. Such programs are particularly valuable for patients in rural or under-resourced regions who may lack access to hospital-based nutrition services.

At the institutional and policy levels, GLIM-based implementation offers strategic advantages by enabling the tracking of nutrition-related quality indicators. Institutions can standardize malnutrition documentation, monitor intervention rates, and align nutritional care with performance-based metrics. Additionally, the formalization of GLIM as a diagnostic construct supports its potential integration into coding systems and reimbursement frameworks, which may increase administrative buy-in and sustainability of services [45].

Nevertheless, disparities in access to GLIM-based care persist. Patients in remote or underserved settings may face challenges in accessing trained dietitians or diagnostic tools such as BIA or CT scans [39]. To address this, care models are increasingly incorporating flexible delivery mechanisms, including remote assessments, simplified diagnostic criteria, and decentralized data collection methods. These innovations ensure that the benefits of GLIM-based care are equitably distributed across patient populations, regardless of geography or institutional capacity.

The GLIM criteria not only facilitate early detection and clinical intervention but also strengthen the infrastructure for ongoing, patient-centered nutritional support. Through enhanced engagement, systematized education, and integrated follow-up services, GLIM-based care contributes to improved outcomes, higher patient satisfaction, and a more holistic approach to oncologic treatment. Moreover, caregiver involvement in nutrition assessment remains an underexplored area. Future research should examine how GLIM-based protocols can engage not only patients but also family members or caregivers in the nutritional care process.

Challenges and future directions

Despite the clinical value and expanding adoption of the GLIM criteria, several challenges continue to hinder their consistent and widespread implementation in gastrointestinal oncology. These barriers span across diagnostic complexity, professional training, institutional infrastructure, policy alignment, and health equity, all of which must be addressed to ensure long-term sustainability and impact [27,35,46,47].

Technological barriers

One of the primary barriers is the complexity of the diagnostic process itself. The GLIM criteria require the simultaneous assessment of phenotypic and etiologic components, necessitating access to advanced diagnostic tools such as computed tomography (CT), DXA, or BIA to evaluate muscle mass [40,48]. While these technologies provide objective and reproducible data, they are not uniformly available across institutions, especially in community hospitals or low-resource settings. Even when available, proper use and interpretation often require specialized training that may not be standard among healthcare providers.

Interpreting the etiologic criterion of inflammation adds further complexity. Cancer patients often exhibit low-grade chronic inflammation due to tumor burden, chemotherapy, or comorbidities, making it difficult to determine whether systemic inflammation contributes meaningfully to nutritional decline. Standard inflammatory markers such as C-reactive protein (CRP) or albumin may not sufficiently differentiate between acute and chronic processes, and cutoff values for GLIM-relevant interpretation remain inconsistently applied in clinical practice [49].

Training gaps and professional exposure

Another substantial limitation lies in the lack of widespread training and clinical exposure to the GLIM criteria among healthcare professionals. Dietitians, physicians, and nurses may receive limited or no formal instruction on GLIM-related assessment methods during their training, resulting in uneven familiarity and inconsistent application [27,50,51]. Without a common foundational understanding, interdisciplinary collaboration is hindered, and the diagnostic process can become fragmented.

Successful implementation often depends on institutional investment in structured training programs and the appointment of “clinical champions” who lead and sustain education and integration efforts [52].

Policy and system integration

At the systems level, the absence of standardized operational pathways presents another critical barrier. While the GLIM criteria provide a consensus on what constitutes malnutrition, they do not prescribe specific workflows for screening, assessment, documentation, or follow-up. As a result, implementation strategies vary widely across institutions, limiting the ability to benchmark performance or track quality metrics [47]. Furthermore, the lack of integration into international diagnostic coding systems, such as ICD-11 (11th revision of the International Classification of Diseases), hampers efforts to link malnutrition diagnoses to reimbursement or quality reporting, thereby weakening institutional incentives for adoption [53]. Although direct financial data are limited, aligning GLIM-based malnutrition diagnosis with coding and reimbursement systems may improve resource allocation and reduce healthcare costs through earlier and more targeted nutritional intervention [53]. The key advantages and barriers associated with GLIM implementation in gastrointestinal oncology are summarized in Table 1.

**Table 1:** Key Advantages and Barriers to GLIM Criteria Implementation in Gastrointestinal Oncology CT, computed tomography; BIA, bioelectrical impedance analysis; GLIM, Global Leadership Initiative on Malnutrition; ICD, International Classification of Diseases.

Advantages	Barriers
Standardized malnutrition diagnosis	Limited access to body composition tools (CT, BIA)
Multidisciplinary workflows and team engagement	Inconsistent GLIM training across professionals
Integration into electronic health records	Absence of reimbursement or ICD coding frameworks
Early patient engagement and self-management	Access disparities in rural or low-resource settings

Equity of implementation remains an urgent concern. Underserved populations, including those in rural areas or in health systems with limited resources, may be disproportionately affected by barriers to GLIM application. These patients often face reduced access to registered dietitians, fewer diagnostic resources, and lower digital infrastructure for telehealth or remote monitoring [35]. Without targeted strategies, GLIM-based frameworks risk reinforcing existing disparities in nutritional care delivery. Notably, multiple studies have validated the predictive value of GLIM-defined malnutrition in hospitalized patients with gastrointestinal cancers. These studies report significant associations between GLIM-based nutritional diagnosis and increased rates of postoperative complications, longer hospital stays, delayed recovery, and decreased overall survival [13,15,16,35,40]. These findings support the clinical relevance of applying GLIM criteria in inpatient oncology settings and emphasize the importance of systematic screening during hospitalization.

Future directions and strategic solutions

Addressing these multifactorial challenges requires coordinated action across clinical, institutional, and policy domains. At the clinical level, the development and validation of surrogate markers for muscle mass and inflammation, such as mid-arm circumference or handgrip strength, may offer accessible alternatives to BIA or CT in primary care and outpatient settings [49]. Workflow adaptations that embed GLIM criteria into EHRs through automated prompts and standardized documentation fields can streamline diagnostic decision-making and promote consistent application [27].

Institutionally, the creation of adaptable GLIM-based protocols for different care contexts, such as surgical oncology, outpatient chemotherapy, or palliative care, would enhance feasibility. Interdisciplinary training modules, especially those that simulate real-world diagnostic scenarios, can accelerate clinician uptake and foster interprofessional understanding [52].

At the policy level, recognizing GLIM-defined malnutrition as a reimbursable condition would significantly incentivize implementation. Coding system integration, performance-based reimbursement, and quality benchmarking could drive greater institutional investment in structured nutritional care models [51,53]. Additionally, policies that support the expansion of dietitian roles and telehealth infrastructure would help extend GLIM-based care to underserved populations.

Finally, future research should continue to evaluate the long-term outcomes of GLIM implementation, including survival rates, treatment adherence, quality of life, and healthcare utilization metrics. Multicenter studies that compare implementation strategies across different health systems and populations would yield insights into best practices and adaptable models [47].

## Conclusions

The implementation of the GLIM criteria has the potential to transform nutritional care in gastrointestinal cancer management. Although originally established as a diagnostic tool, the GLIM criteria have driven broader changes in clinical workflows, interprofessional collaboration, and patient-centered care. Its structured approach reinforces the role of dietitians, promotes earlier nutritional interventions, and supports continuity of care throughout the treatment course. However, challenges persist, including complexity in diagnostic application, variability in training, and gaps in system integration. Addressing these barriers through streamlined workflows, interdisciplinary education, and supportive policy frameworks will be essential for sustainable implementation. Future research should continue to explore how GLIM-based interventions affect long-term outcomes, such as survival, quality of life, and healthcare efficiency.

Ultimately, the GLIM criteria provide not only a standardized method for malnutrition diagnosis but also a foundation for advancing equitable, integrated, and proactive nutrition care in oncology. Looking forward, the GLIM criteria represent more than a diagnostic tool; it is a foundation for sustainable, evidence-based nutrition care. Its continued validation, adaptation, and integration into diverse care environments will be critical for improving outcomes and reducing disparities in nutritional support for patients with gastrointestinal cancers.
